# Interplay of Tumor Morphology and Biology with Postoperative Complications: Prognostic Implications After Resection of Colorectal Liver Metastasis

**DOI:** 10.1245/s10434-026-19229-5

**Published:** 2026-02-18

**Authors:** Abdullah Altaf, Miho Akabane, Kathleen Tong, Mujtaba Khalil, Zayed Rashid, Shahzaib Zindani, Azza Sarfraz, Areesh Mevawalla, Andrea Ruzzenente, Luca Aldrighetti, Irinel Popescu, Minoru Kitago, George Poultsides, Kazunari Sasaki, Federico Aucejo, Itaru Endo, Timothy M. Pawlik

**Affiliations:** 1 Department of Surgery, Division of Surgical Oncology, The Urban Meyer III and Shelley Meyer Chair for Cancer Research, The Ohio State University Wexner Medical Center and James Comprehensive Cancer Center, Columbus, OH USA; 2 Department of Surgery, University of Verona, Verona, Italy; 3 Department of Surgery, San Raffaele Hospital, Milan, Italy; 4 Department of Surgery, Fundeni Clinical Institute, Bucharest, Romania; 5 Department of Surgery, Keio University, Tokyo, Japan; 6 Department of Surgery, Stanford University School of Medicine, Stanford, CA USA; 7 Department of Surgery, Cleveland Clinic Foundation Main Campus, Cleveland, OH USA; 8 Department of Surgery, Yokohama City University School of Medicine, Yokohama, Japan

**Keywords:** Colorectal liver metastasis, Liver resection, Tumor biology, Postoperative complications, Recurrence-free survival, Overall survival

## Abstract

**Objective:**

We sought to evaluate the differential prognostic impact of postoperative complications on recurrence-free survival (RFS) and overall survival (OS) relative to clinicopathologic and clinical biologic indicators associated with colorectal liver metastases (CRLM).

**Methods:**

Patients who underwent curative-intent resection for CRLM between 2000 and 2023 were identified from an international, multi-institutional database. The Genetic And Morphological Evaluation (GAME) score was utilized to risk stratify patients based on KRAS status, carcinoembryonic antigen levels, primary tumor lymph node metastasis, tumor burden score, and extrahepatic disease. The comprehensive complication index (CCI) was used to categorize postoperative complications.

**Results:**

Among 887 patients included in the analytic cohort, the overall incidence of postoperative morbidity was 22.3% (*n *= 197), with 18.8% (*n *= 167) of patients having a high CCI (≥ 26.2). Overall, on multivariable Cox regression analysis, high CCI was independently associated with worse RFS (hazard ration [HR] 1.45 [95% confidence interval {CI} 1.18 – 1.78]; *p *= 0.001) but not with OS (HR 1.14 [95% CI 0.98 – 1.27]; *p *= 0.075). Among patients with a low GAME score, high CCI was associated with both worse RFS (HR 2.17 [95% CI 1.55 – 3.03]; *p *= 0.001) and worse OS (HR1.78 [95% CI 1.22 – 2.58]; *p *= 0.001). In contrast, among patients with medium- and high-risk GAME scores, high CCI was associated with neither RFS (HR 0.97 [95% CI 0.70 – 1.34]; *p *= 0.540) nor OS (HR 0.99 [95% CI 0.67 – 1.46]; *p *= 0.290).

**Conclusion:**

Postoperative complications had a variable prognostic impact on patients undergoing resection for CRLM depending on the severity of the complication and the overall biologic risk of the underlying disease. Patients with more favorable underlying tumor biology were more likely to have adverse oncologic long-term outcomes after experiencing severe postoperative complications.

**Supplementary Information:**

The online version contains supplementary material available at 10.1245/s10434-026-19229-5.

Colorectal cancer is the third most common cancer and second leading cause of cancer-related mortality worldwide.^1,2^ Prognosis depends on several factors, including tumor staging, inflammatory biomarkers, and comorbid conditions, with liver metastasis commonly encountered in advanced stages of disease.^3–5^ Surgical resection remains the primary curative approach for colorectal liver metastases (CRLM).^6,7^ Of note, the introduction of local ablative techniques and effective downsizing treatment options in recent years has markedly expanded surgical candidacy for CRLM.^8–10^ However, expanding surgical indications and more aggressive conversion therapy may increase the risk of postoperative morbidity.^11^ As Dr. Blake Cody once famously noted, “biology is the king”,^12^ and surgical resection is most likely to benefit patients with favorable tumor biology.^13^ To this end, Margonis et al.^14^ developed the preoperative Genetic And Morphological Evaluation (GAME) score by incorporating clinicopathologic and clinically available biologic indicators, including KRAS (Kirsten rat sarcoma viral oncogene homolog) status, carcinoembryonic antigen (CEA) levels, primary tumor lymph node metastasis, tumor burden score (TBS), and presence of extrahepatic disease to inform treatment selection of patients with CRLM.

There is emerging evidence of a complex interplay between tumor biology, surgical morbidity, and long-term outcomes among patients with CRLM.^15^ Postoperative complications such as biliary fistula, liver failure, and sepsis pose substantial challenges in the perioperative management of CRLM.^16^ In addition, major abdominal surgery triggers a systemic inflammatory response marked by elevated cytokine levels.^17–19^ Perioperative complications may exacerbate this inflammatory response and induce immunosuppression, potentially impairing native tumor surveillance and negatively impacting long-term survival outcomes.^19,20^ High postoperative morbidity has generally been associated with worse recurrence-free survival (RFS) and overall survival (OS) among patients undergoing surgical resection for CRLM,^20,21^ but Wang et al.^15^ reported that overall, major complications were not associated with worse OS and RFS. In a similar study on perihilar cholangiocarcinoma, Kawakatsu et al.^22^ demonstrated that—although surgical resection was often associated with high postoperative morbidity—the impact on survival was relatively minor compared with the influence of tumor characteristics. Therefore, understanding the interplay between tumor morphology, biology, and postoperative complications is essential for improving preoperative risk stratification, guiding surgical decision-making, and optimizing postoperative management in patients with CRLM.

In the current study, we sought to evaluate the differential prognostic impact of postoperative complications on RFS and OS relative to the GAME score among patients undergoing surgery for CRLM. The Comprehensive Complication Index (CCI), which quantifies all postoperative complications on a continuous scale from 0 to 100, was utilized to provide a detailed assessment of the total burden of postoperative morbidity. By classifying postoperative complications using the CCI score and tumor biology characterized by the GAME score, we sought to provide a more comprehensive understanding of the perioperative factors that affect long-term survival and oncologic outcomes among patients with CRLM.

## Materials and Methods

### Study Population and Exclusion Criteria

An international, multi-institutional database comprising data from eight major hepatobiliary institutions worldwide was queried to identify patients who underwent curative-intent liver resection for CRLM between 2000 and 2023 (*n *= 3389): Cleveland Clinic Foundation Main Campus, Cleveland, OH, USA (*n *= 120; 13.5%), Fundeni Clinical Institute, Bucharest, Romania (*n *= 91; 10.3%), Northwell Health, New York, NY, USA (*n *= 117; 13.2%), The Ohio State University Wexner Medical Center, Columbus, OH, USA (*n *= 126; 14.2%), Stanford University School of Medicine, Stanford, CA, USA (*n *= 145; 16.3%), University of Verona, Verona, Italy (*n *= 151; 17.0%), and Yokohama City University School of Medicine, Yokohama, Japan (*n *= 137; 15.4%). Patients who received only ablation (*n *= 8; 0.2%), underwent palliative resection (*n *= 19; 0.6%) or two-staged hepatectomy (*n *= 64; 1.9%), had grossly positive surgical resection margins (R2 resection) (*n *= 169; 5.0%), or died within 90 days of surgery (*n *= 54; 1.6%) were excluded. Additionally, patients with missing data on postoperative complications (*n *= 1509; 44.5%), with factors included in the GAME score except KRAS status (*n *= 607; 17.9%), or who were lost to follow-up (*n *= 72; 2.1%) were excluded. Patients with unknown KRAS status were classified as KRAS wild-type to preserve cohort size, a decision supported by prior datasets, and all analyses were validated with a sensitivity analysis excluding these patients. The current study received approval from the institutional review board of each participating institution and adhered to the Transparent Reporting of a Multivariable Prediction Model for Individual Prognosis or Diagnosis (TRIPOD) guidelines.^23^

### Patient Characteristics, Definitions, and Outcomes

Data on patient demographics, including age, sex, race/ethnicity, body mass index, and the Charlson Comorbidity Index score were obtained from standardized datasheets at each institution. Primary tumor characteristics included location of the tumor (colon vs. rectum), preoperative CEA levels, lymph node metastasis, T status, and tumor grade. Similarly, for CRLM characteristics, data were extracted on tumor number, maximum tumor diameter, synchronous versus metachronous liver metastasis (synchronous CRLM was defined as liver metastasis that was detected at the time of or before the primary tumor diagnosis), KRAS status (wild vs. mutated), surgical approach (open vs. minimally invasive surgery [MIS]), tumor grade, tumor location (unilateral vs. bilateral), extent of resection (minor or major), and receipt of neoadjuvant and adjuvant chemotherapy.^15^

The TBS for CRLM combined maximum tumor diameter and number of tumors and was calculated using the formula ${\mathrm{TBS}}^{{2}} = \, \left( {\text{maximum tumor diameter}} \right)^{{2}} + \, \left( {\text{number of tumors}} \right)^{{2}}$.^24^ According to Couinaud’s classification, a major hepatic resection was defined as the surgical resection of more than three segments of the liver.^25^ An R0 resection was characterized by complete absence of macroscopic or microscopic disease at the surgical resection margins.^26^ The definitions for tumor (T) and lymph node (N) status by the American Joint Committee on Cancer (AJCC) were guided by the 8th edition of the AJCC guidelines.^27^ Tumor grade was characterized as well differentiated, moderately differentiated, poorly differentiated, and undifferentiated. The GAME score for each patient was calculated based on KRAS status, CEA levels, primary tumor lymph node metastasis, TBS, and extrahepatic disease (Supplementary Table 1). Patients were classified as low risk (GAME 0–1 points), medium risk (GAME 2–3), and high risk (GAME ≥4).^14^

Postoperative complications, graded according to the Clavien–Dindo classification (CDC), included any adverse event occurring up to 90 days after surgery.^28^ Major postoperative complications were classified as CDC grade ≥ 3 complications. Infectious complications encompassed surgical site, urinary tract, respiratory, catheter-related bloodstream infections, abscess, and systemic sepsis. For each patient, the CCI score was computed on the 90th day after surgery; the CCI quantifies cumulative postoperative morbidity on a continuous scale from 0 to 100, where a score of 100 signifies the patient's death.^29^ Each CDC grade was assigned a specific CCI value and a complication weight (Supplementary Table 2), and the total CCI score was calculated using equation 1:

1 $${\text{CCI }} = \frac{{\sqrt {wC1 + wC2 + wC3 \ldots + wCx} }}{2}$$

A cutoff of 26.2, in line with existing literature, was used to classify patients as low versus high CCI.^15^

Patients from each participating institution received regular monitoring post-resection. Serum tumor markers and radiologic assessments such as abdominal ultrasonography, computed tomography, and magnetic resonance imaging were used to monitor CRLM recurrence. Follow-up visits were generally scheduled every 3–4 months for the first 3 years, biannually for the 4th and 5th years, and annually after that. Recurrence was characterized as suspicious findings on imaging or a histologically proven tumor recurrence. RFS and OS were defined as time elapsed between the date of liver resection and date of CRLM recurrence or date of death and time elapsed between the date of resection and date of death or date of last follow-up, respectively.

### Statistical Analyses

Univariable and multivariable Cox proportional hazards models were employed to predict long-term RFS and OS, presenting results as hazard ratios (HRs) with 95% confidence intervals (CIs). The models incorporated CCI and clinicopathologic variables, including characteristics of both primary tumor and CRLM, previously recognized as confounding factors that can affect prognosis in patients with CRLM. In the multivariable models, factors were incorporated using a backward selection approach, setting the significance threshold at *p*<0.050. The initial analysis was conducted across the entire cohort, with subsequent stratified analysis based on GAME score groups (low risk vs. medium/high risk), to investigate the varying prognostic impact of CCI on RFS and OS as influenced by tumor biology and morphology. A sensitivity analysis was performed that included patients who died within 90 days of surgery to evaluate how early postoperative mortality influenced OS across GAME score categories. A separate sensitivity analysis was performed by restricting the cohort to patients with known KRAS status, given the extent of missing data, to verify consistency of results. Kaplan–Meier survival curves were plotted for RFS and OS, followed by comparisons using the log-rank test.

Categorical variables were presented as frequencies and percentages (%) and analyzed using either the chi-squared test or Fisher’s exact test, as appropriate. Continuous variables were reported as medians with interquartile ranges (IQRs) and compared using the Mann–Whitney U test. Missing values were imputed using the multiple imputation by chained equations technique.^30^ All statistical analyses were two-tailed, and a *p*-value of < 0.05 was considered statistically significant. All statistical analyses were conducted using Python version 3.11 in Visual Studio Code version 1.84.2, and Stata version 18.0 (StataCorp).

## Results

### Patient Characteristics and Postoperative Outcomes

A total of 887 patients who underwent curative-intent surgical resection for CRLM met inclusion criteria and were included in the final analytic cohort (Table 1). Median patient age was 61.1 years (IQR 54.0 – 68.0), and most patients were male (*n* = 531; 59.9%) and White (*n*=702; 79.1%). Approximately one-third of patients had a Charlson Comorbidity Index score > 8 (*n*=284; 32.0%).

**Table 1:** Clinicodemographic characteristics and postoperative complications in the analytic cohort and comparison between patients in Genetic and morphological evaluation (GAME) low-, medium- and high-risk groups

Variables	All patients	Low-risk	Medium-risk	High-risk	*P*-value
	(*n*=887)	(*n*=339; 38.2%)	(*n*=467; 52.7%)	(*n*=81; 9.1%)	
Age, years	61.1 (54.0–68.0)	63.0 (55.0–68.0)	61.0 (54.0–67.0)	61.0 (54.0–66.0)	0.624
*Sex*					0.017
Male	531 (59.9)	219 (64.6)	273 (58.5)	39 (48.1)	
Female	356 (40.1)	120 (35.4)	194 (41.5)	42 (51.9)	
*Race*					0.033
White	702 (79.1)	285 (84.1)	352 (75.4)	65 (80.2)	
Black	22 (2.5)	5 (1.5)	14 (3.0)	3 (3.7)	
Asian	129 (14.5)	44 (13.0)	75 (16.1)	10 (12.3)	
Hispanic	21 (2.4)	2 (0.6)	18 (3.9)	1 (1.2)	
Other	13 (1.5)	3 (0.9)	8 (1.7)	2 (2.5)	
*Charlson comorbidity score*					0.029
≤8	603 (68.0)	213 (62.8)	330 (70.7)	60 (74.1)	
>8	284 (32.0)	126 (37.2)	137 (29.3)	21 (25.9)	
BMI, kg/m^2^	26.7 (24.0–28.4)	26.9 (24.7–28.3)	26.6 (23.6–28.4)	26.5 (24.8–28.5)	0.444
*Primary tumor characteristics*					
Location					0.265
Colon	627 (70.7)	229 (67.6)	340 (72.8)	58 (71.6)	
Rectum	260 (29.3)	110 (32.4)	127 (27.2)	23 (28.4)	
CEA, ng/dL	8.6 (3.2-32.8)	4.7 (2.3-8.7)	16.4 (4.7-58.9)	27.0 (7.0-88.5)	0.038
Lymph node metastasis	578 (65.2)	108 (31.9)	390 (83.5)	80 (98.8)	<0.001
*T status*					0.001
I/II	269 (30.3)	131 (38.6)	140 (30.0)	17 (21.0)	
III/IV	618 (69.7)	208 (61.4)	327 (70.0)	62 (79.0)	
Extrahepatic metastasis	76 (8.6)	0 (0.0)	23 (4.9)	53 (65.4)	<0.001
*Tumor grade*					0.124
Well/moderate	809 (91.2)	317 (93.5)	421 (90.1)	71 (87.7)	
Poorly/undifferentiated	78 (8.8)	22 (6.5)	46 (9.9)	10 (12.3)	
*CRLM characteristics*					
*Liver metastasis*					0.124
Metachronous	372 (41.9)	131 (38.6)	200 (42.8)	41 (50.6)	
Synchronous	515 (58.1)	208 (61.4)	267 (57.2)	40 (49.4)	
Tumor number	2.0 (1.0–3.0)	1.0 (1.0–2.0)	3.0 (1.0–3.0)	3.0 (1.0–4.0)	<0.001
Maximum tumor diameter, cm	2.5 (1.7–3.0)	2.0 (1.3–2.5)	2.5 (2.0–3.3)	2.5 (2.4–3.6)	<0.001
Tumor burden score	3.7 (2.4–4.2)	2.6 (2.1–3.9)	3.9 (3.2–4.6)	3.9 (3.8–5.8)	<0.001
*KRAS status*					<0.001
Wild	250 (28.2)	151 (44.5)	96 (20.6)	3 (3.7)	
Mutated	100 (11.3)	16 (4.7)	65 (13.9)	19 (23.5)	
Not available	537 (60.5)	172 (50.7)	306 (65.5)	59 (72.8)	
Preoperative chemotherapy	400 (45.1)	167 (49.3)	197 (42.2)	36 (44.4)	0.136
*Surgical approach*					0.010
Open	762 (85.9)	299 (88.2)	387 (82.9)	76 (93.8)	
MIS	125 (14.1)	40 (11.8)	80 (17.1)	5 (6.2)	
Major resection	185 (20.9)	58 (15.8)	103 (22.1)	24 (29.6)	0.029
*Tumor grade*					0.022
Well/moderate	846 (95.4)	315 (92.9)	452 (96.8)	79 (97.5)	
Poorly/undifferentiated	41 (4.6)	24 (7.1)	15 (3.2)	2 (2.5)	
*Tumor location*					0.219
Unilobar	621 (70.0)	228 (67.3)	331 (70.9)	62 (76.5)	
Bilobar	266 (30.0)	111 (32.7)	136 (29.1)	19 (23.5)	
*Resection margin*					0.215
R0	761 (85.8)	297 (87.6)	399 (85.4)	65 (80.2)	
R1	126 (14.2)	42 (12.4)	68 (14.6)	16 (19.8)	
Adjuvant chemotherapy	648 (73.1)	257 (75.8)	329 (70.4)	62 (76.5)	0.181
Major complications	115 (13.0)	33 (9.7)	69 (14.8)	13 (16.0)	0.040
Infectious complications	140 (15.8)	61 (18.0)	67 (14.3)	12 (14.8)	0.363
*CCI*					0.292
<26.2	720 (81.2)	282 (83.2)	370 (79.2)	68 (84.0)	
≥26.2	167 (18.8)	57 (16.8)	97 (20.8)	13 (16.0)	

Data are presented as median (interquartile range) or *n* (%) unless otherwise indicated

BMI, Body mass index; CCI, Comprehensive Complication Index; CEA, Carcinoembryonic antigen; CRLM, Colorectal liver metastases; GAME, Genetic And Morphological Evaluation score; KRAS, Kirsten rat sarcoma viral oncogene homolog; MIS, Minimally invasive surgery

The majority of patients had the primary tumor located in the colon (*n *= 627; 70.7%) and had T3 or T4 disease (*n *= 618; 69.7%). Median preoperative CEA levels were 8.6 ng/dL (IQR 3.2 – 32.8), lymph node metastasis was present in 65.2% (*n *= 578) of patients, and extrahepatic metastasis was observed in 8.6% (*n *= 76). Most patients were diagnosed with synchronous liver metastasis (*n *= 515; 58.1%), median TBS of CRLM was 3.7 (IQR 2.4 – 4.2), and 30.0% of the patients had bilateral disease (*n *= 266). KRAS was mutated in 11.3% of patients (*n *= 100). Notably, 45.1% (*n *= 400) of patients received preoperative chemotherapy for CRLM.

Overall, 85.9% of patients (*n *= 762) had an open surgical resection for CRLM; 20.9% of patients underwent major hepatectomy (*n *= 185). R0 resection was achieved in 85.8% (*n*=761) of patients. In the postoperative setting, 334 patients experienced a complication, for an overall morbidity incidence of 37.6%; among these individuals, 15.8% (*n *= 140) of patients experienced an infectious complication. Overall, 13.0% (*n *= 115) of patients experienced a major (CDC grade ≥3) postoperative complication. In total, 18.8% of patients (*n *= 167) had a CCI of ≥26.2. Following surgery, 648 (73.1%) patients received adjuvant chemotherapy.

Based on the GAME score, 38.2% of patients (*n *= 339) were classified as low risk, 52.7% (*n *= 467) as medium risk, and 9.1% (*n *= 81) as high risk (Table 1). In addition to KRAS status, CEA levels, primary tumor lymph node metastasis, TBS, and extrahepatic disease, other differences observed among patients with different GAME scores included patient sex, race, Charlson Comorbidity Index score, and primary tumor T status and tumor grade of CRLM (all *p*<0.05). Of note, patients with a higher GAME score were more likely to experience a major postoperative complication (low risk: 9.7% vs. medium risk: 14.8% vs. high risk: 16.0%; *p*=0.040). Patients with a low GAME score had the best RFS and OS, followed by individuals with a medium-risk GAME score; patients with a high-risk GAME score had the worse RFS and OS outcomes (Supplementary Figures 1a and b). Clinicodemographic characteristics among patients with CCI < 26.2 versus ≥ 26.2 were generally comparable (Supplementary Table 3).

### Overall Prognostic Impact of Postoperative Complications

With a median follow-up duration of 48.2 months (95% CI 42.4 – 55.2), patients with a high CCI had worse 1 year (49.4% [95% CI 41.3 – 57.0] vs. 64.9% [95% CI 61.0 – 68.5]), 3 year (19.0% [95% CI 12.9 – 25.9] vs. 37.9% [95% CI 33.6 – 42.1]) and 5 year (14.2% [95% CI 8.7 – 20.9] vs. 30.8% [95% CI 26.4 – 35.4]) RFS than patients with a low CCI (*p *< 0.001) (Supplementary Figure 2a). Similarly, patients with a high CCI also had worse 1 year (92.5% [95% CI 87.1 – 95.7] vs. 94.1% [95% CI 91.1 – 95.7]), 3 year (55.0% [95% CI 46.0 – 63.0] vs. 65.1% [95% CI 60.5 – 69.3]) and 5 year (32.5% [95% CI 24.0 – 41.3] vs. 46.2% [95% CI 40.9 – 51.3]) OS versus patients with a low CCI (*p *= 0.015) (Supplementary Figure 2b).

On multivariable Cox regression analysis, several factors were associated with worse RFS, including patient-level factors (i.e. older age), primary tumor characteristics (i.e. CEA > 20 ng/dL, T3 or T4 tumor, N1/N2 lymph node status, and poorly or undifferentiated tumor grade), and CRLM characteristics (i.e. extrahepatic disease, mutated KRAS status, higher TBS, preoperative chemotherapy, bilateral disease, R1 resection margin, and adjuvant chemotherapy) (all *p *< 0.05) (Supplementary Table 4). After controlling for these competing risk factors, a high CCI ≥ 26.2 remained independently associated with worse RFS in the overall cohort (reference: CCI < 26.2, HR 1.45 [95% CI 1.18 – 1.78]; *p *= 0.001). In contrast, a high CCI ≥ 26.2 was not associated with OS after adjusting for confounding clinicopathological factors (reference: CCI < 26.2, HR 1.14 [95% CI 0.98 – 1.27]; *p *= 0.075) (Supplementary Table 5). Rather, factors independently associated with worse OS included primary tumor characteristics (i.e., CEA > 20 ng/dL, N1/N2 lymph node status, and poorly or undifferentiated tumor grade) and CRLM characteristics (i.e., extrahepatic disease, mutated KRAS status) (all *p *< 0.05).

### Differential Prognostic Impact of Postoperative Complications Relative to GAME Score

**Fig. 1:**
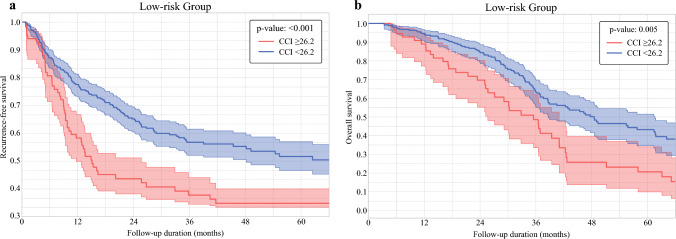
**a** Kaplan–Meier curves for recurrence-free survival in low-risk GAME group stratified by Comprehensive Complication Index (CCI): ≥ 26.2 versus < 26.2. **b** Kaplan–Meier curves for overall survival in low-risk GAME group stratified by Comprehensive Complication Index (CCI): ≥ 26.2 versus < 26.2

Subsequently, stratified analyses based on the GAME score (low risk vs. medium/high risk) were conducted to investigate the differential impact of postoperative complications on RFS and OS relative to tumor biology and morphology. Among patients with a low GAME score (*n* = 339; 38.2%), patients with a high CCI had a worse 1 year (58.6% [95% CI 49.8 – 77.1] vs. 77.4% [95% CI 81.4 – 73.3]), 3 year (37.2% [95% CI 33.3 – 40.0] vs. 56.7% [95% CI 52.8 – 61.1]) and 5 year (34.6% [95% CI 33.2 – 40.1] vs. 51.8% [95% CI 46.7 – 56.8]) RFS versus patients with a low CCI (*p *< 0.001) (Fig. 1a). Similarly, this subset of patients also had worse 1 year (87.2% [95% CI 74.9 – 93.7] vs. 94.6% [95% CI 90.8 – 96.8]), 3 year (46.1% [95% CI 31.3 – 59.7] vs. 62.8% [95% CI 54.9 – 69.8]) and 5 year (20.6% [95% CI 9.9 – 34.0] vs. 43.2% [95% CI 34.6 – 51.5]) OS after experiencing major versus minor postoperative morbidity (*p *= 0.005) (Fig. 1b). On multivariable Cox regression analysis, a high CCI remained independently associated with worse RFS (reference: CCI < 26.2, HR 2.17 [95% CI 1.55 – 3.03]; *p *= 0.001) and OS (reference: CCI < 26.2, HR 1.78 [95% CI 1.22 – 2.58]; *p*=0.005) among patients with a low GAME score (Table 2).

**Table 2:** Univariable and multivariable Cox regression analyses for recurrence-free and overall survival in low-risk Genetic and morphological evaluation (GAME) group

Variables	Recurrence-free survival				Overall survival			
	Univariate analysis		Multivariate analysis		Univariate analysis		Multivariate analysis	
	HR (95% CI)	*P*-value	HR (95% CI)	*P*-value	HR (95% CI)	*P*-value	HR (95% CI)	*P*-value
Age, years	1.02 (1.00–1.03)	0.012	1.02 (1.00–1.03)	0.019	1.01 (0.99–1.03)	0.111		
*Sex* Male Female	Ref. 1.08 (0.80–1.44)	0.608			Ref. 1.18 (0.84–1.66)	0.337		
*Year of surgery* 2000–2010 2011–2023	Ref. 1.05 (0.84–1.23)	0.349			Ref. 0.95 (0.90–1.23)	0.225		
*Charlson Comorbidity Score* ≤8 >8	Ref. 1.05 (0.78–1.42)	0.718			Ref. 1.19 (0.84–1.69)	0.333		
*Primary tumor characteristics*								
*Location* Colon Rectum	Ref. 1.32 (0.99–1.76)	0.059			Ref. 0.95 (0.67–1.36)	0.800		
CEA >20 ng/dL	1.40 (1.05–1.87)	0.021	1.28 (0.96–1.72)	0.090	1.34 (1.01–1.79)	0.042	1.31 (0.98–1.75)	0.065
*T status* I/II III/IV	Ref. 2.35 (1.52–3.62)	< 0.001	Ref. 2.09 (1.35–3.25)	0.001	Ref. 3.40 (1.94–5.98)	< 0.001	Ref. 2.66 (1.31–5.40)	0.007
*N status* N0 N1/N2 Nx	Ref. 1.28 (0.88–1.87) 1.19 (0.62–2.31)	0.190 0.597			Ref. 2.83 (1.64–4.89) 1.54 (0.65–3.65)	< 0.001 0.322	Ref. 1.86 (1.03–3.35) 1.92 (0.77–4.85)	0.039 0.162
*Tumor grade* Well/moderately differentiated Poorly/undifferentiated	Ref. 1.97 (1.19–3.25)	0.008	Ref. 1.50 (0.87–2.56)	0.141	Ref. 2.37 (1.35–4.17)	0.003	Ref. 2.46 (1.38–4.38)	0.002
*CRLM characteristics*								
*Liver metastasis* Metachronous Synchronous	Ref. 1.14 (0.85–1.54)	0.365			Ref. 1.02 (0.72–1.43)	0.927		
Tumor burden score	1.08 (1.03–1.14)	0.001	1.05 (1.02–1.09)	0.013	1.05 (0.99–1.11)	0.091		
Extrahepatic disease	1.58 (1.10–2.26)	0.012	1.41 (0.96–2.07)	0.079	1.72 (1.15–2.57)	0.008	1.63 (1.08–2.46)	0.019
*KRAS status* Wild Mutated Not available	Ref. 1.32 (1.01–1.73) 1.12 (0.87–1.43)	0.044 0.120	1.24 (0.94–1.63)	0.120	Ref. 1.45 (1.09–1.93) 1.34 (0.69–2.21)	0.010 0.245	1.39 (1.03–1.88)	0.033
Preoperative chemotherapy	1.20 (0.90–1.59)	0.206			0.94 (0.66–1.32)	0.649		
*Tumor grade* Well/moderately differentiated Poorly/undifferentiated	Ref. 2.49 (1.54–4.01)	<0.001	Ref. 1.86 (1.10–3.11)	0.020	Ref. 1.22 (0.65–2.30)	0.538		
*Tumor location* Unilobar Bilobar	Ref. 1.73 (1.29–2.32)	<0.001	Ref. 1.26 (0.92–1.72)	0.152	Ref. 1.47 (1.04–2.10)	0.031	Ref. 1.21 (0.84–1.74)	0.314
Hepatic resection Minor Major	Ref. 1.08 (0.79–1.49)	0.609			Ref. 1.24 (0.86–1.78)	0.236		
*Resection margin* R0 R1	Ref. 1.35 (0.94–1.95)	0.101			Ref. 0.92 (0.58–1.46)	0.730		
*Comprehensive complication index* <26.2 ≥26.2	Ref. 2.55 (1.86–3.49)	< 0.001	Ref. 2.17 (1.55–3.03)	0.001	Ref. 1.97 (1.37–2.83)	< 0.001	Ref. 1.78 (1.22–2.58)	0.005
Adjuvant chemotherapy	0.82 (0.60–1.15)	0.252			1.02 (0.66–1.57)	0.919		

CI, Confidence interval; CRLM, Colorectal liver metastases; GAME, Genetic and morphological evaluation score; HR, Hazard ratio

**Fig. 2:**
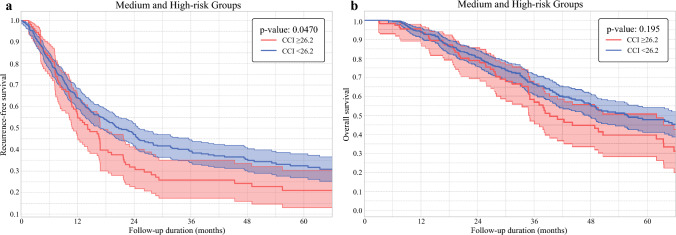
**a** Kaplan–Meier curves for recurrence-free survival in medium- and high-risk GAME groups stratified by Comprehensive Complication Index (CCI): ≥ 26.2 versus < 26.2. **b** Kaplan–Meier curves for overall survival in medium and high-risk GAME groups stratified by Comprehensive Complication Index (CCI): ≥ 26.2 versus < 26.2

Among patients with a medium or high GAME score (*n* = 548; 61.8%), CCI had a marginal effect on 1 year, 3 year, and 5 year RFS versus patients (*p *= 0.05) (Fig. 2a). In addition, there was no difference in OS among patients with a high versus low CCI (*p *= 0.195) (Fig. 2b). On multivariable Cox regression analysis, CCI was not associated with either RFS (reference: CCI < 26.2, HR 0.97 [95% CI 0.70–1.34]; *p *= 0.540) or OS (reference: CCI < 26.2, HR 0.99 [95% CI 0.67 – 1.46]; *p *= 0.290) among patients with a medium or high GAME score (Table 3). The sensitivity analysis including 90-day mortality cases demonstrated consistent findings, with high CCI remaining associated with worse OS only among patients with low GAME scores (Supplementary Tables 6 and 7). Similarly, sensitivity analysis restricting the analytic cohort to patients with known KRAS status likewise demonstrated consistent findings (Supplementary Tables 8 and 9).

**Table 3:** Univariable and multivariable Cox regression analyses for recurrence-free and overall survival in medium and high-risk Genetic and morphological evaluation (GAME) groups

Variables	Recurrence-free survival				Overall survival			
	Univariate analysis		Multivariate analysis		Univariate analysis		Multivariate analysis	
	HR (95% CI)	*P*-value	HR (95% CI)	*P*-value	HR (95% CI)	*P*-value	HR (95% CI)	*P*-value
Age, years	1.01 (0.99–1.02)	0.181			1.01 (0.99–1.02)	0.397		
*Sex* Male Female	Ref. 1.04 (0.83–1.30)	0.725			Ref. 1.10 (0.84–1.45)	0.471		
*Year of surgery* 2000–2010 2011–2023	Ref. 0.85 (0.76–1.08)	0.245			Ref. 0.92 (0.85–1.12)	0.152		
*Charlson comorbidity score* ≤8 >8	Ref. 1.11 (0.87–1.42)	0.390			Ref. 1.46 (1.09–1.94)	0.010	Ref. 1.33 (0.98–1.80)	0.064
*Primary tumor characteristics*								
*Location* Colon Rectum	Ref. 1.01 (0.78–1.29)	0.945			Ref. 1.36 (1.02–1.81)	0.034	Ref. 1.38 (1.03–1.84)	0.030
CEA >20 ng/Dl	1.34 (0.67–1.52)	0.210			1.10 (0.91–1.74)	0.085		
*T status* I/II III/IV	Ref. 1.48 (1.17–1.88)	0.001	Ref. 1.48 (1.16–1.88)	0.002	Ref. 1.68 (1.26–2.23)	< 0.001	Ref. 2.61 (1.27–5.37)	0.009
*N status* N0 N1/N2 Nx	Ref. 1.20 (0.92–1.57) 1.20 (0.90–1.59)	0.181 0.213			Ref. 1.61 (1.16–2.22) 1.03 (0.72–1.46)	0.004 0.881	Ref. 1.49 (1.12–2.45) 1.24 (0.86–1.77)	0.022 0.243
*Tumor grade* Well/moderately differentiated Poorly/undifferentiated	Ref. 1.33 (0.95–1.86)	0.093			Ref. 1.19 (0.80–1.77)	0.392		
*CRLM characteristics*								
*Liver metastasis* Metachronous Synchronous	Ref. 1.08 (0.86–1.36)	0.484			Ref. 0.91 (0.70–1.19)	0.517		
Tumor burden score	1.06 (1.03–1.10)	0.001	1.04 (1.00–1.08)	0.034	1.05 (1.02–1.10)	0.006	1.03 (0.99–1.07)	0.168
Extrahepatic disease	1.64 (1.20–2.24)	0.002	1.53 (1.09–2.13)	0.014	2.71 (1.75–4.18)	< 0.001	2.45 (1.58–3.81)	0.005
KRAS status Wild Mutated Not available	Ref. 1.30 (1.05–1.62) 1.54 (0.72–1.98)	0.015 0.320	1.18 (0.91–1.54)	0.210	1.41 (1.11–1.79) 0.98 (0.76–1.93)	0.005 0.320	1.34 (1.02–1.76)	0.037
Preoperative chemotherapy	1.42 (1.13–1.77)	0.002	0.76 (0.60–0.95)	0.018	1.01 (0.76–1.32)	0.964		
*Tumor grade* Well/moderately differentiated Poorly/undifferentiated	Ref. 1.57 (0.90–2.75)	0.109			Ref. 2.08 (1.18–3.65)	0.011	Ref. 1.72 (0.96–3.06)	0.066
*Tumor location* Unilobar Bilobar	Ref. 1.53 (1.20–1.95)	< 0.001	Ref. 1.48 (1.15–1.91)	0.002	Ref. 1.41 (1.06–1.89)	0.020	Ref. 1.22 (0.90–1.67)	0.189
*Hepatic resection* Minor Major	Ref. 1.06 (0.80–1.41)	0.663			Ref. 0.80 (0.56–1.14)	0.228		
*Resection margin* R0 R1	Ref. 1.80 (1.34–2.42)	< 0.001	Ref. 1.54 (1.14–2.09)	0.005	Ref. 1.09 (0.70–1.68)	0.702		
*Comprehensive complication index* <26.2 ≥26.2	Ref. 1.22 (1.01–1.54)	0.040	Ref. 0.97 (0.70–1.34)	0.540	Ref. 0.99 (0.67–1.46)	0.290		
Adjuvant chemotherapy	0.71 (0.56–0.90)	0.006	0.64 (0.50–0.83)	0.001	1.38 (0.99–1.92)	0.059		

CI, confidence interval; CRLM, Colorectal liver metastases; GAME, Genetic and morphological evaluation score; HR, Hazard ratio

## Discussion

Colorectal cancer remains one of the most common malignancies worldwide.^1,2^ Despite advances in systemic and surgical therapies, CRLM are still associated with poor long-term outcomes, with a reported long-term cure of only 14%.^31^ Accurate postoperative prognostication is essential for selecting surgical candidates, optimizing systemic therapy, and tailoring surveillance strategies. While postoperative complications have been reported to affect long-term survival after hepatectomy for CRLM adversely,^21,32^ the extent to which this impact on prognosis may vary according to tumor morphology and biology remains poorly understood. The current study was, therefore, important as we utilized the GAME score—a composite risk model integrating tumor morphology (via TBS) and tumor biology (KRAS mutation, CEA levels, lymph node status, and extrahepatic disease)—to stratify patients and investigate the differential prognostic impact of postoperative complications.^14^ Our findings indicate that the prognostic effect of postoperative complications varies according to both complication severity and underlying biological risk. Specifically, patients with favorable tumor biology, as reflected by a low GAME score, experienced increased risk of recurrence and worse survival in the presence of severe complications. In contrast, among patients with intermediate- or high-risk GAME scores, severe complications did not impact either RFS or OS. These findings highlight the relative importance that complications can have on oncologic outcomes based on underlying tumor biology. Patients with favorable tumor biology appear disproportionately vulnerable to the adverse effects of severe postoperative complications.

Previous studies have suggested that postoperative complications may adversely impact long-term outcomes after hepatectomy for CRLM.^21,32^ Notably, a recent study by Wang et al.^15^ applied the Modified Clinical Score (M-CS) to stratify patients and demonstrated that severe complications were associated with worse RFS and OS among patients with low M-CS, whereas such an association was not observed among high-risk patients. The M-CS was proposed by Brudvik et al.^33^ based on the earlier Clinical Risk Score proposed by Fong et al.^34^ and incorporates three key variables: RAS mutation status, tumor size, and lymph node involvement. Although the M-CS improves on the original Clinical Risk Score, it captures tumor burden only through tumor size and excludes tumor number, an important morphological component. Tumor biology and tumor burden may directly modulate the oncologic consequences of postoperative complications. To address these gaps, the GAME score was developed, incorporating TBS—a composite measure derived from tumor size and number using a Pythagorean formula—to better reflect tumor burden.^14,24^ In addition, the GAME score includes preoperative CEA level, which has been widely recognized as a surrogate for tumor biology and aggressiveness, as well as the presence of extrahepatic disease, which has become increasingly accepted as not an absolute contraindication to CRLM resection strategies.^35^ Collectively, the five factors included in the GAME score provide a more comprehensive and biologically meaningful preoperative risk stratification model. Importantly, the GAME score outperforms the Fong score and can be readily applied preoperatively to inform treatment selection.^14^

In the current study, severe postoperative complications—defined as a CCI ≥ 26.2—were independently associated with worse RFS, but not with OS, in the overall cohort. These data suggested that complications may primarily contribute to increased recurrence risk rather than directly influencing mortality. Importantly, while prior literature has established the general association between postoperative morbidity and poor long-term outcomes, our study demonstrates that this effect is not homogenous across all patients. Instead, postoperative complications appear to have a disproportionately adverse impact on patients with biologically favorable tumors, effectively neutralizing their otherwise improved prognosis. Conversely, in patients with aggressive tumor biology, long-term outcomes remain poor regardless of complication status, likely due to dominant biological risk. Prior studies suggest that the inflammatory response triggered by complications—characterized by elevated cytokines and growth factors—may promote recurrence by supporting metastatic outgrowth or accelerating micrometastatic disease.^36,37^ Although the exact mechanisms remain incompletely understood, it has been hypothesized that postoperative immune suppression and systemic inflammation may underlie the adverse oncologic effects of complications.^38^ Wang et al.^15^ reported that the CCI was a more sensitive predictor of prognosis than either infectious complications or Clavien–Dindo grade III or higher complications. This finding may be due to the ability of the CCI to more accurately capture the cumulative burden of all postoperative complications, including those of lower grades; in fact, CCI has been correlated with a patient’s overall physiological status.^29,39^ Indeed, a high CCI may compromise recovery and limit tolerance to adjuvant therapies, thereby contributing to worse long-term outcomes. In line with these findings, the current study confirmed the prognostic utility of the CCI in the context of CRLM resection, particularly as a predictor of recurrence.

Of note, we observed that the detrimental effect of a high CCI varied significantly depending on biological risk, as stratified by the GAME score. Among patients with low GAME scores—indicating favorable tumor biology—severe complications were associated with worse RFS and OS. In contrast, among patients with intermediate or high GAME scores, CCI had no impact on long-term outcomes. This pattern likely reflects a ceiling effect in which poor tumor biology dominates prognosis in patients with a high GAME score, diminishing the relative influence of complications. In contrast, among patients who might have otherwise been expected to have better outcomes (i.e., low GAME scores), complications and downstream sequalae may induce worse cancer-specific outcomes. This phenomenon—in which a covariate loses significance in high-risk strata due to dominant competing risks—has been described in prior statistical literature.^40^ These findings reaffirm that tumor biology is the dominant determinant of long-term outcomes in CRLM. However, among biologically favorable patients, postoperative complications may act as a critical modifiable factor capable of reversing an otherwise favorable prognosis, highlighting a distinct window for meaningful clinical intervention in this subgroup. Of note, although postoperative complications did not reach statistical significance among patients with intermediate- or high-risk GAME scores, this likely reflects the predominance of adverse tumor biology and competing risks rather than a lack of clinical relevance. Although complications have a less pronounced impact on long-term survival in patients with intermediate- or high-risk GAME scores, they can still impair recovery, delay systemic therapy, and negatively affect quality of life, underscoring the ongoing importance of minimizing postoperative morbidity for all patients. Our findings underscore that while biologically favorable patients are disproportionately affected, perioperative optimization remains essential across all GAME groups.

The clinical implications of the current findings are twofold. First, among patients with favorable tumor biology—as indicated by a low GAME score—prevention of postoperative complications is of paramount importance. Among these individuals, complications may serve as modifiable prognostic factors, directly undermining an otherwise favorable oncologic trajectory. The findings of the current study suggest that postoperative morbidity is not simply a universal prognostic factor but a biologically conditional one, reinforcing the need to direct perioperative optimization efforts. This insight challenges the assumption that all patients are equally affected by postoperative morbidity and supports a more tailored approach to risk mitigation. The recognition that postoperative complications can negate the survival advantage conferred by favorable tumor biology highlights the need to prioritize complication prevention in biologically low-risk patients. Recent advancements such as MIS approach, refined intraoperative strategies, Enhanced Recovery After Surgery protocols, and perioperative interventions, including nutritional support and inflammation-modulating interventions, have all contributed to reduce the incidence of complications.^41,42^ Of note, patients who underwent MIS were less likely to have a high CCI, with the MIS approach being more common among patients with a low than a high CCI (15.4% vs. 6.8%, *p* = 0.014). Additionally, for patients with high GAME scores, whose disease biology already predicts poor outcomes, postoperative morbidity should not be the deciding factor in determining whether surgery is appropriate. In this context, the GAME score offers a pragmatic and accessible tool for risk stratification, as it relies entirely on routinely available preoperative data. Given its simplicity and clinical relevance, the GAME score has potential for bedside use to guide personalized decision-making regarding surgical planning and postoperative care.

The results of the current study should be interpreted in the light of several limitations. As a retrospective observational analysis, causal inferences cannot be definitively established. Given that a considerable number of patients were excluded because of missing data, the potential for selection bias cannot be fully excluded. Additionally, minor differences in perioperative management protocols across participating centers may have introduced variability that could affect outcomes. The appropriateness of the selected CCI cutoff value, as well as the threshold used for GAME score-based risk group classification, warrants further validation. A notable limitation of the current study is the proportion of patients with unknown KRAS mutation status, largely attributable to the absence of routine molecular profiling in earlier years of the study. These patients were included in the GAME score–based stratified analyses using available clinical documentation, which may have introduced some degree of misclassification. However, we chose to retain them to preserve statistical power, and the final analytic cohort exceeded the sample size of the original GAME study, strengthening the validity of our findings.^14^ The generalizability of the findings across different institutions and patient populations should also be confirmed in external cohorts.

In conclusion, the prognostic impact of postoperative complications following resection of CRLM varied according to both the severity of complications and the underlying tumor biology. Severe postoperative morbidity compromised RFS and OS among patients with favorable tumor biology, as characterized by a low GAME score. These findings emphasize the critical importance of prevention of postoperative complications especially among low-risk patients to optimize long-term oncologic outcomes. Conversely, among patients with high GAME scores, postoperative complications did not influence survival. Data in the current study serve to emphasize the interplay of tumor biology, modifiable surgical factors, and complications and their subsequent immune-modulatory effects on outcomes of patients undergoing resection of CRLM.

## Supplementary Information

Below is the link to the electronic supplementary material.

Supplementary file1 (DOCX 746 KB)
