# Supplementary material for: Interplay of Tumor Morphology and Biology with Postoperative Complications: Prognostic Implications After Resection of Colorectal Liver Metastasis
Source: Ann Surg Oncol. 2026 Feb 18;33(5):3930–42. doi: 10.1245/s10434-026-19229-5 (PMC13083369; doi:10.1245/s10434-026-19229-5)

**Supplementary Table 1.** Calculation of Genetic And Morphological Evaluation (GAME) score.

| GAME Score Predictive Factors | Points |
| --- | --- |
| KRAS mutation | 1 |
| Carcinoembryonic antigen (CEA) ≥20 ng/mL | 1 |
| Primary tumor lymph node metastasis | 1 |
| Tumor burden score (TBS)  3 ≤ TBS < 9  ≥9 | 1  2 |
| Extrahepatic disease | 2 |

**Supplementary Table 2.** Weights of different grades of postoperative complications for calculation of CCI.

| Clavien-Dindo Classification | wC (weight of complication) | CCI Value |
| --- | --- | --- |
| Grade I | 300 | 8.7 |
| Grade II | 1,750 | 20.9 |
| Grade IIIa | 2,750 | 26.2 |
| Grade IIIb | 4,550 | 33.7 |
| Grade IVa | 7,200 | 42.4 |
| Grade IVb | 8,550 | 46.2 |

**^CCI:^** ^Comprehensive Complication Index.^

^Clavien-Dindo Classification V results in CCI 100.^

^CCI =^ $\frac{\sqrt{wC1+wC2+wC3\ldots+wCx}}{2}$

**Supplementary Table 3.** Clinicodemographic characteristics in the analytic cohort and comparison between patients with Comprehensive Complication Index <26.2 vs. ≥26.2.

| Variables | All patients  (n=887) | CCI <26.2  (n=720, 81.2%) | CCI ≥26.2  (n=167, 18.8%) | *P* value |
| --- | --- | --- | --- | --- |
| Age (years) | 61.1 (54.0-68.0) | 61.0 (54.0–67.0) | 61.8 (56.8–68.3) | 0.402 |
| Sex  Male  Female | 531 (59.9%)  356 (40.1%) | 454 (60.1%)  301 (39.9%) | 77 (58.3%)  55 (41.7%) | 0.770 |
| Charlson comorbidity score  ≤8  >8 | 603 (68.0%)  284 (32.0%) | 508 (67.3%)  247 (32.7%) | 95 (72.0%)  37 (28.0%) | 0.335 |
| BMI (kg/m^2^) | 26.7 (24.0-28.4) | 26.7 (24.0–28.4) | 26.6 (23.9–28.3) | 0.767 |
| *Primary tumor characteristics* |  |  |  |  |
| Location  Colon  Rectum | 627 (70.7%)  260 (29.3%) | 536 (71.0%)  219 (29.0%) | 91 (68.9%)  41 (31.1%) | 0.710 |
| CEA (ng/dL) | 8.6 (3.2-32.8) | 8.7 (3.3–33.9) | 6.8 (2.6–21.9) | 0.154 |
| Lymph node metastasis | 578 (65.2%) | 501 (66.4%) | 77 (58.3%) | 0.092 |
| T status  I/II  III/IV | 269 (30.3%)  618 (69.7%) | 238 (31.5%)  517 (68.5%) | 31 (23.5%)  101 (76.5%) | 0.080 |
| Extrahepatic metastasis | 76 (8.6%) | 73 (9.7%) | 3 (2.3%) | 0.009 |
| Tumor grade  Well/moderate  Poorly/undifferentiated | 809 (91.2%)  78 (8.8%) | 693 (91.8%)  62 (8.2%) | 116 (87.9%)  16 (12.1%) | 0.195 |
| *CRLM characteristics* |  |  |  |  |
| Liver metastasis  Metachronous  Synchronous | 372 (41.9%)  515 (58.1%) | 319 (42.3%)  436 (57.7%) | 53 (40.2%)  79 (59.8%) | 0.722 |
| Tumor number | 2.0 (1.0-3.0) | 2.0 (1.0–3.0) | 2.0 (1.0–3.0) | 0.691 |
| Maximum tumor diameter (cm) | 2.5 (1.7-3.0) | 2.2 (1.3–2.9) | 2.5 (1.8–3.0) | 0.007 |
| Tumor burden score | 3.7 (2.4-4.2) | 3.3 (2.3–3.9) | 3.8 (2.5–4.3) | 0.043 |
| KRAS status  Wild  Mutated  Not available | 250 (28.2%)  100 (11.3%)  537 (60.5%) | 215 (28.5%)  86 (11.4%)  454 (60.1%) | 35 (26.5%)  14 (10.6%)  83 (62.9%) | 0.838 |
| Preoperative chemotherapy | 400 (45.1%) | 340 (45.0%) | 60 (45.5%) | 0.895 |
| Surgical approach  Open  MIS | 762 (85.9%)  125 (14.1%) | 639 (84.6%)  116 (15.4%) | 123 (93.2%)  9 (6.8%) | 0.014 |
| Major resection | 185 (20.9%) | 53 (7.0%) | 132 (100.0%) | <0.001 |
| Tumor grade  Well/moderate  Poorly/undifferentiated | 846 (95.4%)  41 (4.6%) | 727 (96.3%)  28 (3.7%) | 119 (90.2%)  13 (9.8%) | 0.004 |
| Tumor location  Unilobar  Bilobar | 621 (70.0%)  266 (30.0%) | 538 (71.3%)  217 (28.7%) | 83 (62.9%)  49 (37.1%) | 0.045 |

^Abbreviations:^ **^BMI^**^: Body mass index,^ **^CEA^**^: Carcinoembryonic antigen,^ **^CRLM^**^: Colorectal liver metastases,^ **^KRAS^**^:^ ^Kirsten rat sarcoma viral oncogene homolog,^ **^CCI^**^: Comprehensive complication index.^

**Supplementary Table 4.** Univariable and multivariable Cox regression analysis for recurrence-free survival in the entire cohort.

| Variables | Univariate analysis | | Multivariate analysis | |
| --- | --- | --- | --- | --- |
|  | HR 95% CI | *P* value | HR 95% CI | *P* value |
| Age (years) | 1.01 (1.00-1.02) | 0.011 | 1.01 (1.00-1.02) | 0.015 |
| Sex  Male  Female | Ref.  1.05 (0.88-1.25) | 0.607 |  |  |
| Charlson comorbidity score  ≤8  >8 | Ref.  1.10 (0.91-1.33) | 0.327 |  |  |
| *Primary tumor characteristics* |  |  |  |  |
| Location  Colon  Rectum | Ref.  1.14 (0.94-1.37) | 0.181 |  |  |
| CEA >20 ng/dL | 1.39 (1.16-1.67) | 0.001 | 1.26 (1.05-1.51) | 0.015 |
| T status  I/II  III/IV | Ref.  1.67 (1.38-2.04) | <0.001 | Ref.  2.61 (1.43-3.47) | <0.001 |
| N status  N0  N1/N2  Nx | Ref.  1.26 (1.02-1.56)  1.19 (0.92-1.53) | 0.033  0.193 | Ref.  1.98 (1.29-3.03)  0.80 (0.63-1.02) | 0.002  0.066 |
| Tumor grade  Well/moderately differentiated  Poorly/undifferentiated | Ref.  1.45 (1.10-1.91) | 0.009 | Ref.  1.36 (1.02-1.82) | 0.038 |
| *CRLM characteristics* |  |  |  |  |
| Liver metastasis  Metachronous  Synchronous | Ref.  1.12 (0.93-1.33) | 0.231 |  |  |
| Extrahepatic disease | 1.58 (1.20-2.08) | 0.001 | 2.21 (1.53-3.19) | 0.001 |
| KRAS status  Wild  Mutated  Not available | Ref.  1.33 (1.08-1.64)  1.15 (0.84-1.57) | 0.007  0.395 | Ref.  1.23 (1.03-1.54)  0.91 (0.72-1.15) | 0.010  0.451 |
| Tumor burden score | 1.07 (1.04-1.10) | <0.001 | 1.03 (1.00-1.06) | 0.046 |
| Preoperative chemotherapy | 1.35 (1.13-1.61) | 0.001 | 0.83 (0.69-0.99) | 0.039 |
| Tumor grade  Well/moderately differentiated  Poorly/undifferentiated | Ref.  2.00 (1.39-2.86) | 0.001 | Ref.  1.41 (0.97-2.04) | 0.069 |
| Tumor location  Unilobar  Bilobar | Ref.  1.61 (1.34-1.94) | <0.001 | Ref.  1.42 (1.16-1.73) | 0.001 |
| Hepatic resection  Minor  Major | Ref.  1.09 (0.88-1.34) | 0.443 |  |  |
| Resection margin  R0  R1 | Ref.  1.61 (1.28-2.03) | <0.001 | Ref.  1.37 (1.08-1.75) | 0.010 |
| Comprehensive complication index  <26.2  ≥26.2 | Ref.  1.61 (1.32-1.97) | <0.001 | Ref.  1.45 (1.18-1.78) | 0.001 |
| Adjuvant chemotherapy | 0.75 (0.62-0.91) | 0.004 | 0.69 (0.57-0.85) | 0.001 |

^Abbreviations:^ **^CEA^**^: Carcinoembryonic antigen,^ **^CRLM^**^: Colorectal liver metastases,^ **^KRAS^**^:^ ^Kirsten rat sarcoma viral oncogene homolog,^ **^HR^**^: Hazards ratio,^ **^CI^**^: Confidence interval.^

**Supplementary Table 5.** Univariable and multivariable Cox regression analysis for overall survival in the entire cohort.

| Variables | Univariate analysis | | Multivariate analysis | |
| --- | --- | --- | --- | --- |
|  | HR 95% CI | *P* value | HR 95% CI | *P* value |
| Age (years) | 1.01 (0.99-1.02) | 0.096 |  |  |
| Sex  Male  Female | Ref.  1.12 (0.91-1.38) | 0.289 |  |  |
| Charlson comorbidity score  ≤8  >8 | Ref.  1.37 (1.10-1.71) | 0.006 | Ref.  1.18 (0.94-1.48) | 0.162 |
| *Primary tumor characteristics* |  |  |  |  |
| Location  Colon  Rectum | Ref.  1.20 (0.96-1.50) | 0.105 |  |  |
| CEA >20 ng/dL | 1.30 (1.04-1.62) | 0.019 | 1.29 (1.03-1.62) | 0.024 |
| T status  I/II  III/IV | Ref.  1.80 (1.41-2.28) | <0.001 | Ref.  1.52 (0.83-2.78) | 0.174 |
| N status  N0  N1/N2  Nx | Ref.  1.89 (1.45-2.47)  1.15 (0.83-1.59) | <0.001  0.382 | Ref.  1.54 (1.21-2.12)  0.89 (0.48-1.66) | 0.008  0.730 |
| Tumor grade  Well/moderately differentiated  Poorly/undifferentiated | Ref.  1.39 (1.01-1.92) | 0.045 | Ref.  1.41 (1.01-1.97) | 0.046 |
| *CRLM characteristics* |  |  |  |  |
| Liver metastasis  Metachronous  Synchronous | Ref.  0.96 (0.78-1.19) | 0.748 |  |  |
| Extrahepatic disease | 1.65 (1.21-2.24) | 0.001 | 2.69 (1.75-4.13) | <0.001 |
| KRAS status  Wild  Mutated  Not available | Ref.  1.63 (1.25-2.13)  1.36 (0.91-2.01) | <0.001  0.126 | Ref.  1.34 (1.09-1.69)  1.23 (0.79-1.89) | 0.036  0.359 |
| Tumor burden score | 1.05 (1.02-1.08) | 0.002 | 1.02 (0.99-1.06) | 0.139 |
| Preoperative chemotherapy | 0.98 (0.79-1.21) | 0.824 |  |  |
| Tumor grade  Well/moderately differentiated  Poorly/undifferentiated | Ref.  1.43 (0.94-2.17) | 0.093 |  |  |
| Tumor location  Unilobar  Bilobar | Ref.  1.43 (1.14-1.78) | 0.002 | Ref.  1.15 (0.90-1.46) | 0.253 |
| Hepatic resection  Minor  Major | Ref.  0.99 (0.77-1.27) | 0.967 |  |  |
| Resection margin  R0  R1 | Ref.  1.02 (0.75-1.41) | 0.874 |  |  |
| Comprehensive complication index  <26.2  ≥26.2 | Ref.  1.23 (1.02-1.39) | 0.008 | Ref.  1.14 (0.98-1.27) | 0.075 |
| Adjuvant chemotherapy | 1.26 (0.96-1.63) | 0.088 |  |  |

^Abbreviations:^ **^CEA^**^: Carcinoembryonic antigen,^ **^CRLM^**^: Colorectal liver metastases,^ **^KRAS^**^:^ ^Kirsten rat sarcoma viral oncogene homolog,^ **^HR^**^: Hazards ratio,^ **^CI^**^: Confidence interval.^

**Supplementary Table 6.** Univariable and multivariable Cox regression analyses for overall survival in low-risk GAME group, including patients with 90-day mortality.

| Variables | Univariate analysis | | Multivariate analysis | |
| --- | --- | --- | --- | --- |
|  | HR 95% CI | *P* value | HR 95% CI | *P* value |
| Age (years) | 1.01 (0.99–1.03) | 0.105 |  |  |
| Sex  Male  Female | Ref.  1.15 (0.83–1.61) | 0.394 |  |  |
| Charlson comorbidity score  ≤8  >8 | Ref.  1.17 (0.83–1.66) | 0.367 |  |  |
| *Primary tumor characteristics* |  |  |  |  |
| Location  Colon  Rectum | Ref.  0.93 (0.65–1.32) | 0.693 |  |  |
| T status  I/II  III/IV | Ref.  3.28 (1.88–5.74) | <0.001 | Ref.  2.50 (1.23–5.07) | 0.010 |
| N status  N0  N1/N2  Nx | Ref.  1.88 (1.04–3.39)  1.54 (0.65-3.65) | 0.037  0.322 | Ref.  1.92 (1.01–3.64)  1.92 (0.77-4.85) | 0.045  0.162 |
| Tumor grade  Well/moderately differentiated  Poorly/undifferentiated | Ref.  2.34 (1.32–4.14) | 0.003 | Ref.  2.38 (1.30–4.36) | 0.005 |
| *CRLM characteristics* |  |  |  |  |
| Liver metastasis  Metachronous  Synchronous | Ref.  1.02 (0.75-1.41) | 0.857 |  |  |
| Tumor burden score | 1.04 (0.98–1.10) | 0.164 |  |  |
| Preoperative chemotherapy | 0.92 (0.66-1.29) | 0.649 |  |  |
| Tumor grade  Well/moderately differentiated  Poorly/undifferentiated | Ref.  1.22 (0.65-2.30) | 0.538 |  |  |
| Tumor location  Unilobar  Bilobar | Ref.  1.44 (1.01–2.07) | 0.045 | Ref.  1.21 (0.82–1.77) | 0.343 |
| Hepatic resection  Minor  Major | Ref.  1.23 (0.85–1.77) | 0.264 |  |  |
| Resection margin  R0  R1 | Ref.  0.90 (0.57–1.43) | 0.662 |  |  |
| Comprehensive complication index  <26.2  ≥26.2 | Ref.  1.94 (1.35–2.80) | <0.001 | Ref.  1.80 (1.21–2.67) | 0.004 |
| Adjuvant chemotherapy | 1.01 (0.65–1.57) | 0.964 |  |  |

^Abbreviations:^ **^GAME^**^: Genetic And Morphological Evaluation score,^ **^CRLM^**^: Colorectal liver metastases,^ **^HR^**^: Hazards ratio,^ **^CI^**^: Confidence interval.^

**Supplementary Table 7.** Univariable and multivariable Cox regression analyses for overall survival in medium and high-risk GAME groups, including patients with 90-day mortality.

| Variables | Univariate analysis | | Multivariate analysis | |
| --- | --- | --- | --- | --- |
|  | HR 95% CI | *P* value | HR 95% CI | *P* value |
| Age (years) | 1.01 (0.99–1.02) | 0.385 |  |  |
| Sex  Male  Female | Ref.  1.08 (0.83–1.42) | 0.558 |  |  |
| Charlson comorbidity score  ≤8  >8 | Ref.  1.40 (1.03–1.91) | 0.031 | Ref.  1.41 (1.03–1.93) | 0.034 |
| *Primary tumor characteristics* |  |  |  |  |
| Location  Colon  Rectum | Ref.  1.32 (1.05-1.74) | 0.030 | Ref.  1.39 (1.09-1.62) | 0.025 |
| T status  I/II  III/IV | Ref.  1.66 (1.24–2.22) | <0.001 | Ref.  2.45 (1.20–5.01) | 0.014 |
| N status  N0  N1/N2  Nx | Ref.  1.46 (1.05–2.03)  1.54 (0.65-3.65) | 0.024  0.322 | Ref.  1.51 (1.09–2.10)  1.92 (0.77-4.85) | 0.035  0.162 |
| Tumor grade  Well/moderately differentiated  Poorly/undifferentiated | Ref.  2.06 (1.17–3.63) | 0.012 | Ref.  2.01 (1.10–3.68) | 0.023 |
| *CRLM characteristics* |  |  |  |  |
| Liver metastasis  Metachronous  Synchronous | Ref.  1.02 (0.72-1.43) | 0.941 |  |  |
| Tumor burden score | 1.04 (1.00–1.08) | 0.061 |  |  |
| Preoperative chemotherapy | 0.92 (0.66-1.29) | 0.649 |  |  |
| Tumor grade  Well/moderately differentiated  Poorly/undifferentiated | Ref.  1.22 (0.65-2.30) | 0.538 |  |  |
| Tumor location  Unilobar  Bilobar | Ref.  1.39 (1.04–1.87) | 0.026 | Ref.  1.18 (0.86–1.63) | 0.303 |
| Hepatic resection  Minor  Major | Ref.  0.79 (0.55–1.13) | 0.198 |  |  |
| Resection margin  R0  R1 | Ref.  0.90 (0.51-1.49) | 0.510 |  |  |
| Comprehensive complication index  <26.2  ≥26.2 | Ref.  1.01 (0.68–1.50) | 0.358 |  |  |
| Adjuvant chemotherapy | 1.28 (0.95-1.92) | 0.089 |  |  |

^Abbreviations:^ **^GAME^**^: Genetic And Morphological Evaluation score,^ **^CRLM^**^: Colorectal liver metastases,^ **^HR^**^: Hazards ratio,^ **^CI^**^: Confidence interval.^

**Supplementary Table 8.** Univariable and multivariable Cox regression analyses for recurrence-free and overall survival in low-risk GAME group among patients with known KRAS status.

| Variables | Recurrence-free Survival | | | | Overall Survival | | | |
| --- | --- | --- | --- | --- | --- | --- | --- | --- |
|  | Univariate analysis | | Multivariate analysis | | Univariate analysis | | Multivariate analysis | |
|  | HR 95% CI | *P* value | HR 95% CI | *P* value | HR 95% CI | *P* value | HR 95% CI | *P* value |
| Age (years) | 1.01 (0.94–1.05) | 0.125 |  |  | 1.01 (0.98–1.04) | 0.142 |  |  |
| Sex  Male  Female | Ref.  1.10 (0.78–1.55) | 0.588 |  |  | Ref.  1.12 (0.79–1.60) | 0.506 |  |  |
| Charlson comorbidity score  ≤8  >8 | Ref.  1.06 (0.75–1.50) | 0.732 |  |  | Ref.  1.18 (0.81–1.73) | 0.360 |  |  |
| *Primary tumor characteristics* |  |  |  |  |  |  |  |  |
| Location  Colon  Rectum | Ref.  1.27 (0.93–1.74) | 0.126 |  |  | Ref.  0.97 (0.67–1.40) | 0.863 |  |  |
| T status  I/II  III/IV | Ref.  2.08 (1.33–3.25) | 0.002 | Ref.  1.91 (1.20–3.06) | 0.006 | Ref.  3.05 (1.71–5.44) | <0.001 | Ref.  2.45 (1.17–5.15) | 0.018 |
| N status  N0  N1/N2  Nx | Ref.  1.22 (0.84–1.80)  1.43 (0.62-2.31) | 0.276  0.797 |  |  | Ref.  2.83 (1.64-4.89)  1.54 (0.65-3.65) | <0.001  0.322 | Ref.  1.78 (0.99–3.20)  1.87 (0.43-4.25) | 0.039  0.159 |
| Tumor grade  Well/moderately differentiated  Poorly/undifferentiated | Ref.  1.83 (1.06–3.15) | 0.028 | Ref.  1.44 (1.03–2.59) | 0.046 | Ref.  2.26 (1.25–4.09) | 0.007 | Ref.  2.30 (1.27–4.18) | 0.006 |
| *CRLM characteristics* |  |  |  |  |  |  |  |  |
| Liver metastasis  Metachronous  Synchronous | Ref.  1.09 (0.79–1.52) | 0.592 |  |  | Ref.  1.01 (0.70–1.47) | 0.941 |  |  |
| Tumor burden score | 1.07 (1.02–1.11) | 0.006 | 1.05 (1.01–1.09) | 0.020 | 1.04 (0.99–1.10) | 0.125 |  |  |
| Preoperative chemotherapy | 1.15 (0.84–1.59) | 0.382 |  |  | 0.94 (0.66–1.35) | 0.752 |  |  |
| Tumor grade  Well/moderately differentiated  Poorly/undifferentiated | Ref.  2.69 (1.84-4.75) | <0.001 | Ref.  2.06 (1.40-4.34) | 0.012 | Ref.  1.43 (0.45-2.10) | 0.432 |  |  |
| Tumor location  Unilobar  Bilobar | Ref.  1.60 (1.17–2.20) | 0.003 | Ref.  1.18 (0.84–1.67) | 0.312 | Ref.  1.39 (0.97–1.99) | 0.071 |  |  |
| Hepatic resection  Minor  Major | Ref.  1.01 (0.59-1.87) | 0.653 |  |  | Ref.  1.32 (0.76-1.85) | 0.326 |  |  |
| Resection margin  R0  R1 | Ref.  1.72 (0.82-2.43) | 0.163 |  |  | Ref.  0.97 (0.75-1.66) | 0.210 |  |  |
| Comprehensive complication index  <26.2  ≥26.2 | Ref.  2.41 (1.65–3.52) | <0.001 | Ref.  2.08 (1.42–3.06) | 0.001 | Ref.  1.87 (1.24–2.82) | 0.003 | Ref.  1.66 (1.10–2.51) | 0.018 |
| Adjuvant chemotherapy | 0.74 (0.62-1.45) | 0.432 |  |  | 0.91 (0.39-1.62) | 0.574 |  |  |

^Abbreviations:^ **^GAME^**^: Genetic And Morphological Evaluation score,^ **^CRLM^**^: Colorectal liver metastases,^ **^HR^**^: Hazards ratio,^ **^CI^**^: Confidence interval.^

**Supplementary Table 9.** Univariable and multivariable Cox regression analyses for recurrence-free and overall survival in medium and high-risk GAME groups among patients with known KRAS status.

| Variables | Recurrence-free Survival | | | | Overall Survival | | | |
| --- | --- | --- | --- | --- | --- | --- | --- | --- |
|  | Univariate analysis | | Multivariate analysis | | Univariate analysis | | Multivariate analysis | |
|  | HR 95% CI | *P* value | HR 95% CI | *P* value | HR 95% CI | *P* value | HR 95% CI | *P* value |
| Age (years) | 1.01 (0.99–1.03) | 0.254 |  |  | 1.01 (0.99–1.03) | 0.437 |  |  |
| Sex  Male  Female | Ref.  1.06 (0.83–1.36) | 0.649 |  |  | Ref.  1.13 (0.86–1.49) | 0.388 |  |  |
| Charlson comorbidity score  ≤8  >8 | Ref.  1.11 (0.87–1.43) | 0.398 |  |  | Ref.  1.46 (1.07–1.97) | 0.016 | Ref.  1.43 (1.05–1.95) | 0.023 |
| *Primary tumor characteristics* |  |  |  |  |  |  |  |  |
| Location  Colon  Rectum | Ref.  1.05 (0.80–1.37) | 0.704 |  |  | Ref.  1.38 (1.03–1.84) | 0.031 | Ref.  1.35 (1.01–1.80) | 0.045 |
| T status  I/II  III/IV | Ref.  1.47 (1.14–1.89) | 0.004 | Ref.  1.45 (1.12–1.88) | 0.006 | Ref.  1.66 (1.21–2.26) | 0.002 | Ref.  2.46 (1.19–5.11) | 0.016 |
| N status  N0  N1/N2  Nx | Ref.  1.20 (0.92–1.58)  1.28 (0.76-1.74) | 0.186  0.353 |  |  | Ref.  1.57 (1.13–2.18)  1.10 (0.68-1.50) | 0.008  0.765 | Ref.  1.49 (1.12-2.45)  1.32 (0.82-1.97) | 0.022  0.341 |
| Tumor grade  Well/moderately differentiated  Poorly/undifferentiated | Ref.  1.28 (0.90–1.82) | 0.160 |  |  | Ref.  1.16 (0.78–1.73) | 0.465 |  |  |
| *CRLM characteristics* |  |  |  |  |  |  |  |  |
| Liver metastasis  Metachronous  Synchronous | Ref.  1.11 (0.67-1.51) | 0.432 |  |  | Ref.  0.72 (0.57-1.32) | 0.437 |  |  |
| Tumor burden score | 1.06 (1.02–1.10) | 0.008 | 1.04 (1.00–1.08) | 0.041 | 1.05 (1.01–1.09) | 0.025 | 1.02 (0.98–1.06) | 0.210 |
| Preoperative chemotherapy | 1.37 (1.08–1.75) | 0.010 | 0.78 (0.62–0.99) | 0.037 | 1.02 (0.77–1.36) | 0.900 |  |  |
| Tumor grade  Well/moderately differentiated  Poorly/undifferentiated | Ref.  1.45 (0.79-2.93) | 0.229 |  |  | Ref.  2.43 (1.32-3.34) | 0.010 | Ref.  1.45 (1.12-3.43) | 0.032 |
| Tumor location  Unilobar  Bilobar | Ref.  1.50 (1.16–1.93) | 0.002 | Ref.  1.46 (1.12–1.90) | 0.005 | Ref.  1.39 (1.03–1.87) | 0.034 | Ref.  1.19 (0.87–1.64) | 0.271 |
| Hepatic resection  Minor  Major | Ref.  1.14 (0.74-1.56) | 0.526 |  |  | Ref.  0.92 (0.65-1.34) | 0.231 |  |  |
| Resection margin  R0  R1 | Ref.  1.71 (1.25–2.33) | 0.001 | Ref.  1.49 (1.09–2.03) | 0.011 | Ref.  1.07 (0.70–1.65) | 0.749 |  |  |
| Comprehensive complication index  <26.2  ≥26.2 | Ref.  1.16 (0.86–1.58) | 0.341 |  |  | Ref.  0.97 (0.65–1.45) | 0.240 |  |  |
| Adjuvant chemotherapy | 0.75 (0.59–0.96) | 0.018 | 0.67 (0.52–0.87) | 0.003 | 1.35 (0.96–1.90) | 0.089 |  |  |

^Abbreviations:^ **^GAME^**^: Genetic And Morphological Evaluation score,^ **^CRLM^**^: Colorectal liver metastases,^ **^HR^**^: Hazards ratio,^ **^CI^**^: Confidence interval.^

**Supplementary Figure 1a.** Kaplan–Meier curves for recurrence-free survival stratified by GAME score: low- vs. medium- vs. high-risk.

**
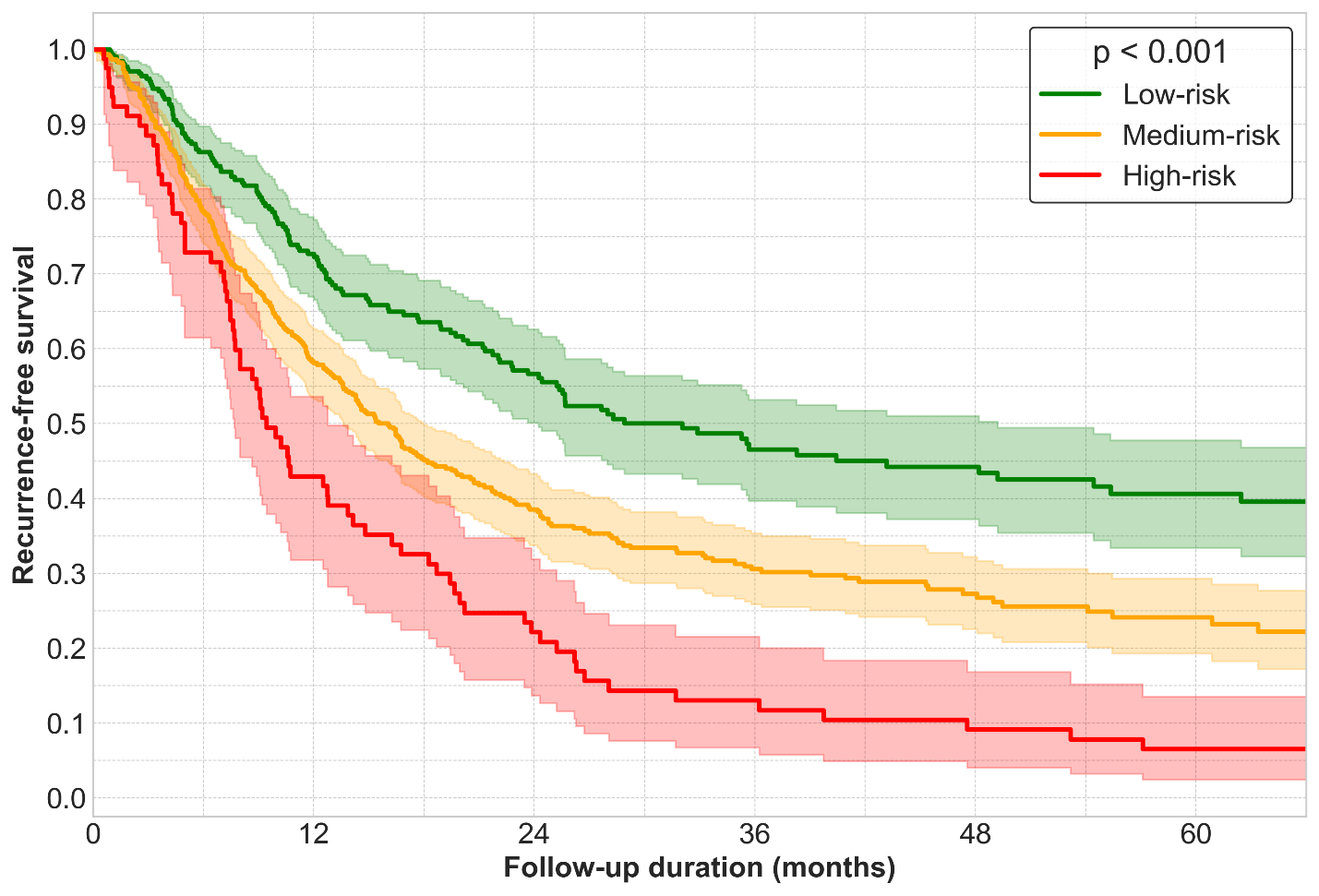
**

**Supplementary Figure 1b.** Kaplan–Meier curves for overall survival stratified by GAME score: low- vs. medium- vs. high-risk.


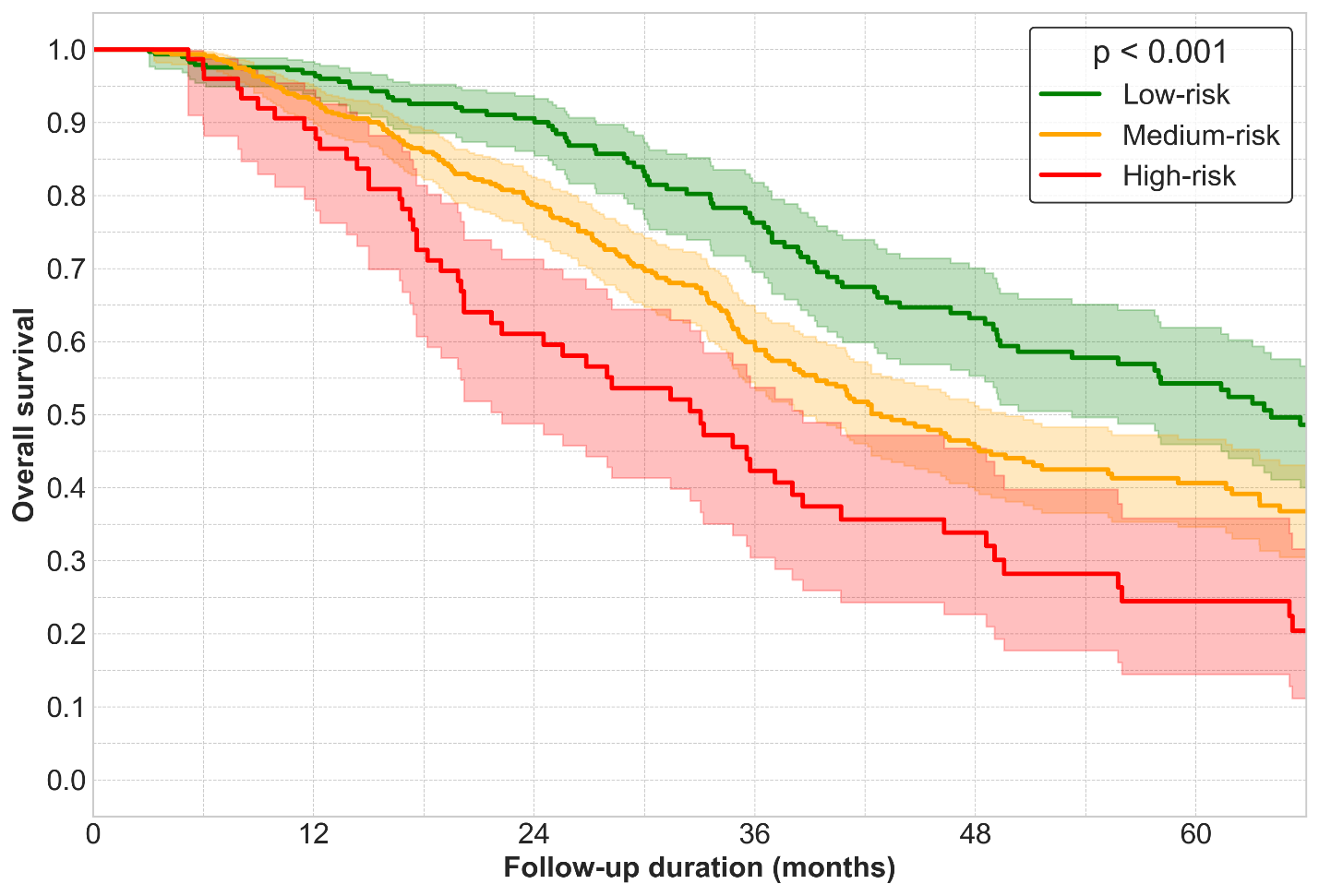


**Supplementary Figure 2a.** Kaplan–Meier curves for recurrence-free survival in the entire cohort stratified by Comprehensive Complication Index (CCI): ≥26.2 versus <26.2.


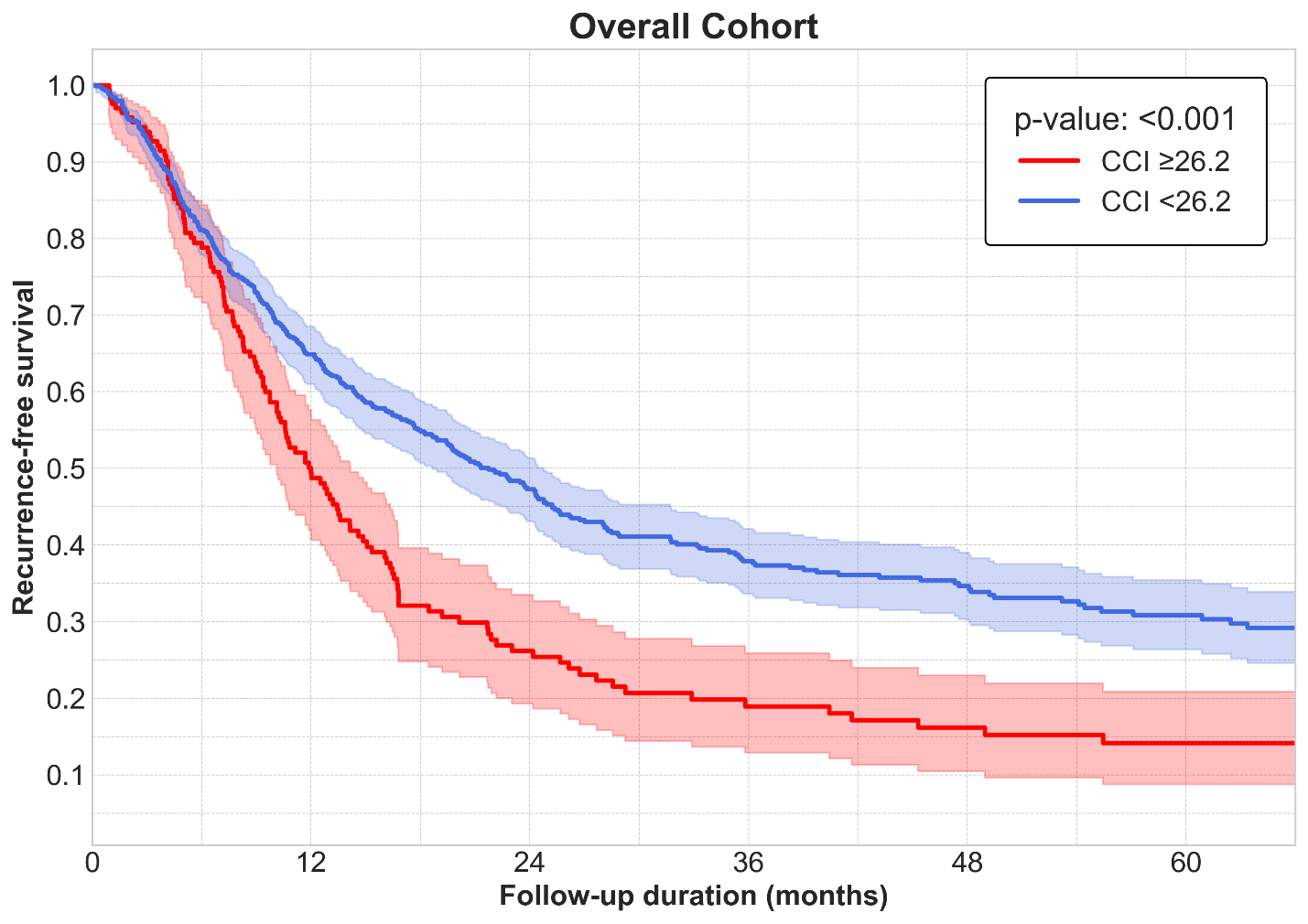


**Supplementary Figure 2b.** Kaplan–Meier curves for overall survival in the entire cohort stratified by Comprehensive Complication Index (CCI): ≥26.2 versus <26.2.


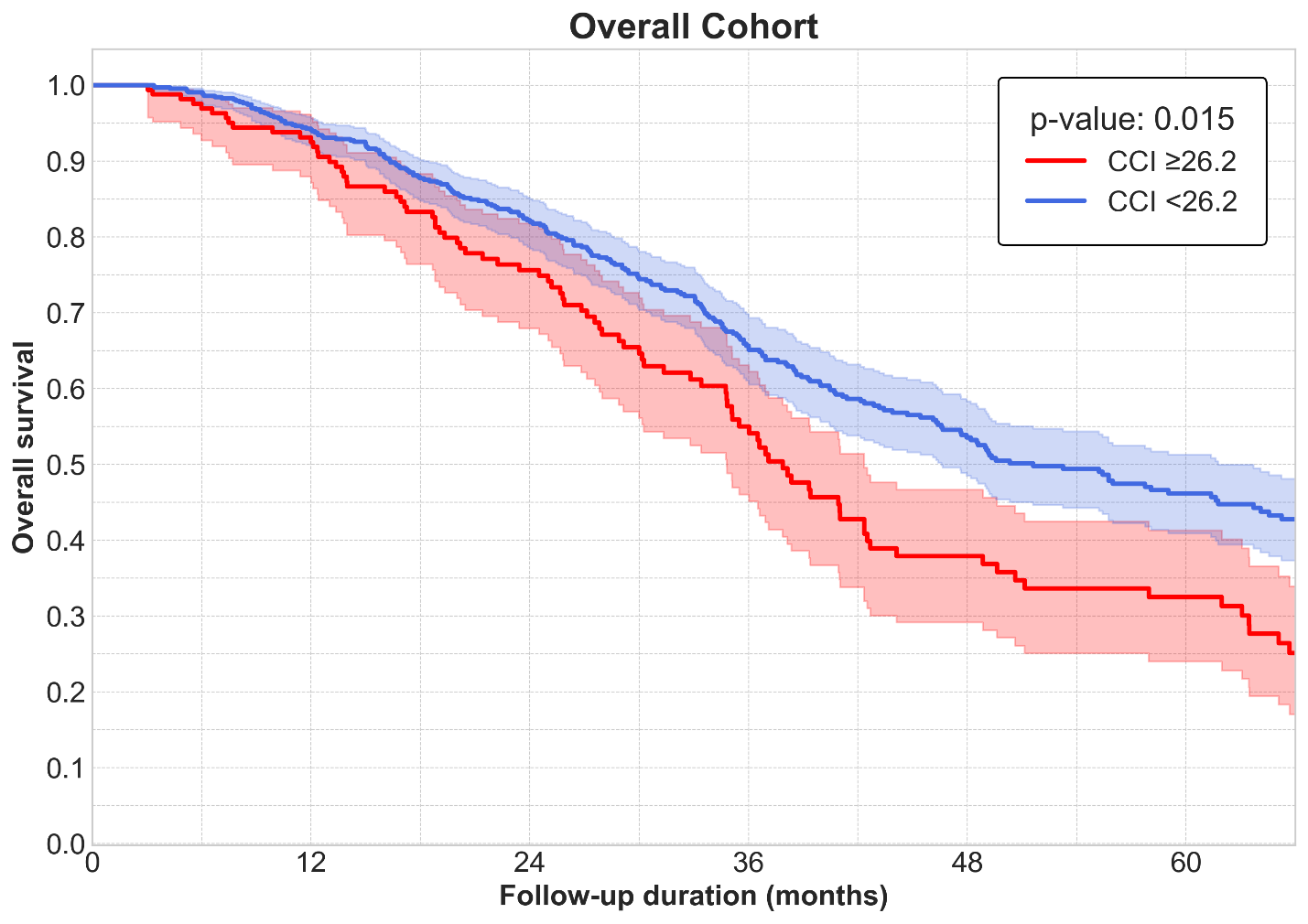

Supplement: Supplementary file 1 — Supplementary file1 (DOCX 746 KB) [file 10434_2026_19229_MOESM1_ESM.docx]
